# Exploring the Therapeutic Potential of Epigallocatechin-3-gallate (Green Tea) in Periodontitis Using Network Pharmacology and Molecular Modeling Approach

**DOI:** 10.3390/ijms26189144

**Published:** 2025-09-19

**Authors:** Balu Kamaraj

**Affiliations:** Department of Dental Education, College of Dentistry, Imam Abdulrahman Bin Faisal University, Dammam 31441, Saudi Arabia; bkranganayaki@iau.edu.sa

**Keywords:** EGCG, periodontitis, network pharmacology, docking, MD simulations, PPI

## Abstract

Periodontitis is a common inflammatory disease affecting the supporting structures of teeth. Epigallocatechin-3-gallate (EGCG), a polyphenol found in green tea, is known for its therapeutic properties in various diseases, including periodontitis. This study aims to identify the gene targets of EGCG and investigate its potential in modulating molecular pathways associated with periodontitis. The potential gene targets of EGCG were obtained from the traditional Chinese medicine systems pharmacology database and analysis platform (TCMSP) and SwissTargetPrediction databases, while genes associated with periodontitis were sourced from GeneCards and Gene Expression Omnibus (GEO) datasets. By overlapping the two datasets, ten common target genes were identified. To explore their functional relevance, enrichment analyses such as Gene Ontology (GO) and REACTOME pathway mapping were conducted. Protein–protein interaction (PPI) networks were then generated, and further analyses involving molecular docking and molecular dynamics (MD) simulations were carried out to evaluate the binding affinity and structural stability of EGCG with the selected target proteins. Ten common genes (MMP2, MMP14, BCL2, STAT1, HIF1A, MMP9, MMP13, VEGFA, ESR1, and PPARG) were identified. PPI network and GO and pathway analyses identified the promising hub genes as ESR1, MMP2, MMP9, MMP13, and STAT1 and which highlighted roles in tissue development, extracellular matrix remodeling, and signaling pathways such as interleukin and matrix metalloproteinase activities. Molecular docking and MD simulations revealed strong binding interactions between EGCG and key proteins (ESR1, MMP2, MMP9, MMP13, and STAT1), with favorable binding energies and stable complexes. Among these, ESR1 and MMP13 exhibited the most favorable docking scores and stability in molecular dynamics simulations and MM–PBSA calculations. This study provides valuable insights into the molecular mechanisms of EGCG in periodontitis treatment. The findings suggest that ESR1 and MMP13 are the most promising targets for EGCG, supported by strong binding interactions and stable conformations in simulations. These results offer a foundation for further experimental studies and potential therapeutic applications of EGCG in managing periodontitis.

## 1. Introduction

Periodontitis is a persistent, multifactorial infectious disorder marked by inflammation in the periodontal tissues, which is primarily driven by the host’s immune response to microbial biofilms on the tooth surface. Periodontitis causes the gradual deterioration of the structures supporting the teeth, resulting in attachment loss and, eventually, tooth loss [1,2]. Globally, approximately 10–15% of adults experience tooth loss due to periodontitis, making it a leading cause of oral health deterioration [3]. Beyond its localized effects, periodontitis has been closely associated with systemic diseases such as diabetes and cardiovascular disease, underscoring its significance in maintaining both oral and systemic health [4,5,6]. Data from the 4th National Oral Health Survey in Mainland China highlight the high prevalence of periodontal diseases, with gingivitis and periodontitis remaining major public health concerns. Alarmingly, the detection rate of dental calculus among individuals aged 35–44 years was 96.7%, and gingival bleeding was observed in 87.4% of this demographic, emphasizing the urgent need for effective prevention and treatment strategies [7].

The primary goal of periodontal treatment is to halt disease progression, restore tissue health, and improve overall oral and systemic health. Conventional treatment methods include basic periodontal therapy (e.g., scaling and root planning), surgical interventions, and adjunctive pharmacotherapy. Among these, pharmacotherapy plays a critical role, particularly in managing acute infections, addressing areas inaccessible to mechanical debridement, and treating patients with systemic conditions or limited oral hygiene capabilities [8]. However, the limitations of current pharmacological agents, including resistance and side effects, have spurred interest in developing alternative therapeutic approaches.

Natural compounds have emerged as promising candidates for periodontal therapy due to their multitarget mechanisms, reduced side effects, and potential to address inflammation, microbial challenges, oxidative stress, and bone resorption. Recent studies have highlighted the therapeutic potential of natural products, including antibacterial, anti-inflammatory, and bone-protective properties, as effective adjuncts in managing periodontal diseases [9]. These findings support the exploration of natural compounds, such as epigallocatechin-3-gallate (EGCG) from green tea, as novel agents in the prevention and treatment of periodontitis [10,11,12,13].

Recent evidence highlights several molecular mediators involved in the pathogenesis of periodontitis namely estrogen receptor 1 (ESR1), matrix metalloproteinases (MMP2, MMP9, MMP13), and signal transducer and activator of transcription 1 (STAT1) [14,15,16,17,18]. ESR1 is significant for periodontal tissue integrity and bone metabolism, with polymorphisms linked to disease susceptibility [14,15]. MMP2, MMP9, and MMP13 are key enzymes responsible for extracellular matrix breakdown and bone resorption, contributing to the progression of periodontal destruction [16,17]. STAT1 regulates immune and inflammatory responses, and its activation is associated with accelerated tissue damage in periodontitis [18]. EGCG is the most abundant catechin in green tea and has been widely studied for its antioxidant, anti-inflammatory, and antimicrobial properties. It has shown efficacy in modulating host immune responses, inhibiting matrix metalloproteinases, and reducing oxidative stress, processes that are critically involved in periodontal tissue destruction [11,12,13]. Despite this evidence, the precise molecular mechanisms by which EGCG exerts its protective effects in periodontitis remain poorly understood, representing a significant research gap.

To uncover the mechanisms of natural compounds, advanced computational methods like network pharmacology and molecular docking are increasingly employed [19,20,21,22,23,24,25,26]. Network pharmacology provides a holistic approach to understanding the multi-target and multi-pathway actions of bioactive molecules, while molecular docking and molecular dynamics simulations offer structural insights into their interactions with key proteins. This integrative strategy has been successfully applied in studies investigating compounds like shikonin, baicalein, and Mori Folium extracts, revealing their potential to regulate critical molecular pathways in periodontitis [23,24,25].

This study focuses on utilizing network pharmacology, molecular docking, and molecular dynamics simulation to explore the potential mechanisms of EGCG in the treatment of periodontitis. By identifying key targets and pathways modulated by EGCG, this research aims to provide a comprehensive understanding of its therapeutic role and contributes to the development of innovative, natural compound-based treatments for periodontal diseases.

## 2. Results

### 2.1. Gene Targets of Epigallocatechin-3-gallate (EGCG)

**Figure 1:**
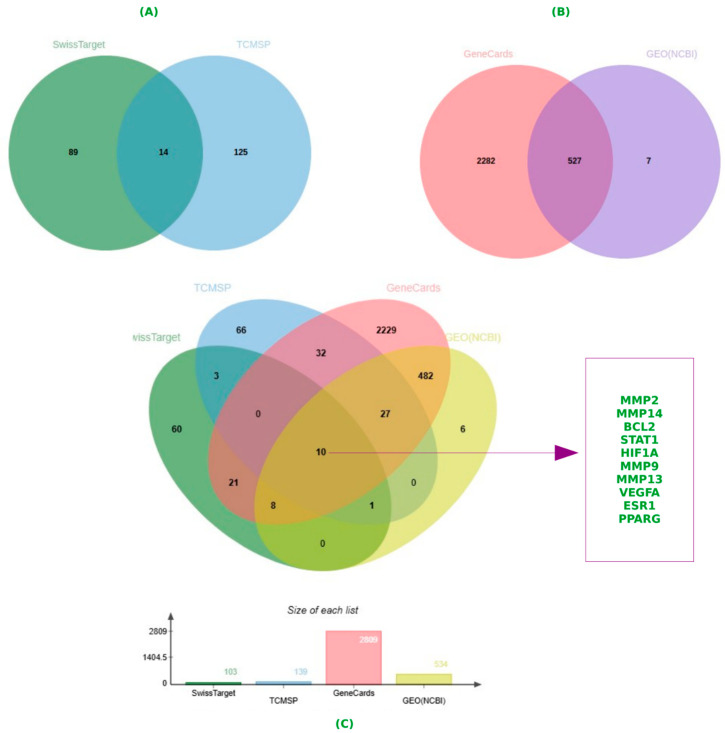
Visualization of intersecting target genes linked to EGCG and periodontitis using a Venn diagram. (**A**) Overlapping EGCG targets from SwissTarget and TCMSP. (**B**) Overlapping periodontitis targets from GeneCards and GEO (NCBI). (**C**) Integrated Venn analysis showing 10 common genes: MMP2, MMP14, BCL2, STAT1, HIF1A, MMP9, MMP13, VEGFA, ESR1 and PPARG. EGCG, epigallocatechin-3-gallate; TCMSP, the traditional Chinese medicine systems pharmacology database and analysis platform; GEO, Gene Expression Omnibus; gene symbols—MMP2, MMP14, BCL2, STAT1, HIF1A, MMP9, MMP13, VEGFA, ESR1 and PPARG.

Using the TCMSP database, 139 targets were identified in relation to EGCG, while SwissTarget Prediction predicted an additional 103 targets. After eliminating duplicates, 14 potential targets were retained for further investigation (Figure 1A and Table S1).

### 2.2. Gene Targets of Periodontitis

Utilizing “periodontitis” as the keyword, 2809 disease-related targets were retrieved from the GeneCards database, while 534 relevant targets were obtained from the GEO database. Integrating the results and removing duplicates resulted in a collection of 527 periodontitis-associated targets (Figure 1B). By analyzing the intersection between the 14 EGCG-associated targets and the 527 periodontitis-related targets, a total of 10 common genes were identified. These shared targets—MMP2, MMP14, BCL2, STAT1, HIF1A, MMP9, MMP13, VEGFA, ESR1, and PPARG—were subsequently utilized for further enrichment analysis and protein–protein interaction (PPI) network construction (Figure 1C).

### 2.3. Enrichment Analysis

**Figure 2:**
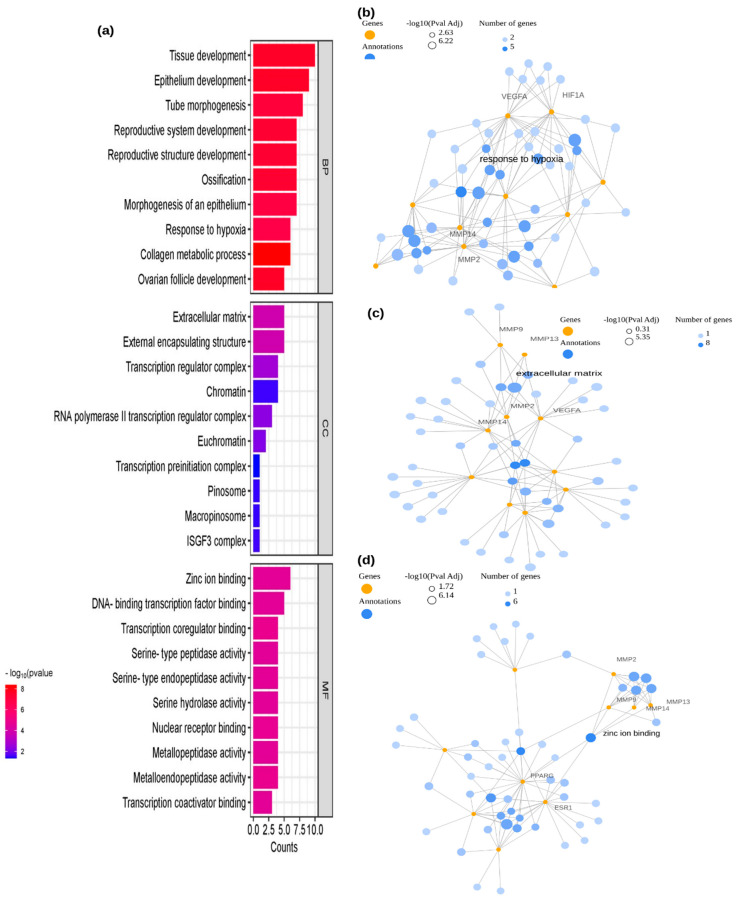
Gene Ontology (GO) enrichment analysis of the top 10 shared target genes between EGCG and periodontitis. (**a**) Enriched terms under the categories of Biological Process (BP), Cellular Component (CC), and Molecular Function (MF). (**b**–**d**) Representative network illustrating BP, CC, and MF terms along with their associated target genes. Color gradients represent varying *p* value thresholds, while bar lengths indicate the number of genes mapped to each term. BP, Biological Process; CC, Cellular Component; MF, Molecular Function; Gene symbols—MMP2, MMP14, BCL2, STAT1, HIF1A, MMP9, MMP13, VEGFA, ESR1 and PPARG.

The shared target genes of EGCG and periodontitis were analyzed using R Shiny GO v0.81 for Gene Ontology (GO), REACTOME & KEGG pathway, and disease enrichment (RGD & Alliance) (Table S2). The GO analysis encompassed three categories: Biological Process (BP), Cellular Component (CC), and Molecular Function (MF). In total, 1172 statistically significant GO terms were identified, including 1000 from BP, 32 from CC, and 140 from MF. The top 10 significantly enriched terms from each category were illustrated in Figure 2a, where the Y-axis represents the *p* value, and the X-axis indicates gene counts. A lower *p* value signifies stronger enrichment of GO terms.

The GO enrichment analysis revealed that the target genes were associated with several critical biological functions. Within the BP category, they played key roles in processes such as tissue development (GO:0009888), epithelium development (GO:0060429), tube morphogenesis (GO:0035239), reproductive system development (GO:0061458), reproductive structure development (GO:0048608), ossification (GO:0001503), epithelium morphogenesis (GO:0002009), response to hypoxia (GO:0001666), collagen metabolic process (GO:0032963), and ovarian follicle development (GO:0001541). Additionally, Figure 2b presents a network visualization of BP-enriched terms, specifically highlighting response to hypoxia and its four key target genes: MMP2, MMP14, HIF1A, and VEGFA. Similarly, within the CC category, the target genes were found to be involved in essential structures such as the extracellular matrix (GO:0031012), external encapsulating structure (GO:0030312), transcription regulator complex (GO:0005667), chromatin (GO:0000785), RNA polymerase II transcription regulator complex (GO:0090575), euchromatin (GO:0000791), transcription preinitiation complex (GO:0097550), pinosome (GO:0044352), macropinosome (GO:0044354), and ISGF3 complex (GO:0070721) (Figure 2a). The network of CC-enriched terms, specifically the extracellular matrix and its five key target genes—MMP2, MMP9, MMP13, MMP14, and VEGFA—is illustrated in Figure 2c. In the MF category, the target genes were involved in crucial molecular functions, including zinc ion binding (GO:0008270), DNA-binding transcription factor binding (GO:0140297), transcription coregulator binding (GO:0001221), serine-type peptidase activity (GO:0008236), serine-type endopeptidase activity (GO:0004252), serine hydrolase activity (GO:0017171), nuclear receptor binding (GO:0016922), metallopeptidase activity (GO:0008237), metalloendopeptidase activity (GO:0004222), and transcription coactivator binding (GO:0001223) (Figure 2a). Furthermore, Figure 2d visualizes the network of MF-enriched terms, particularly zinc ion binding, along with its six key target genes: MMP2, MMP9, MMP13, MMP14, PPARG, and ESR1.

**Figure 3:**
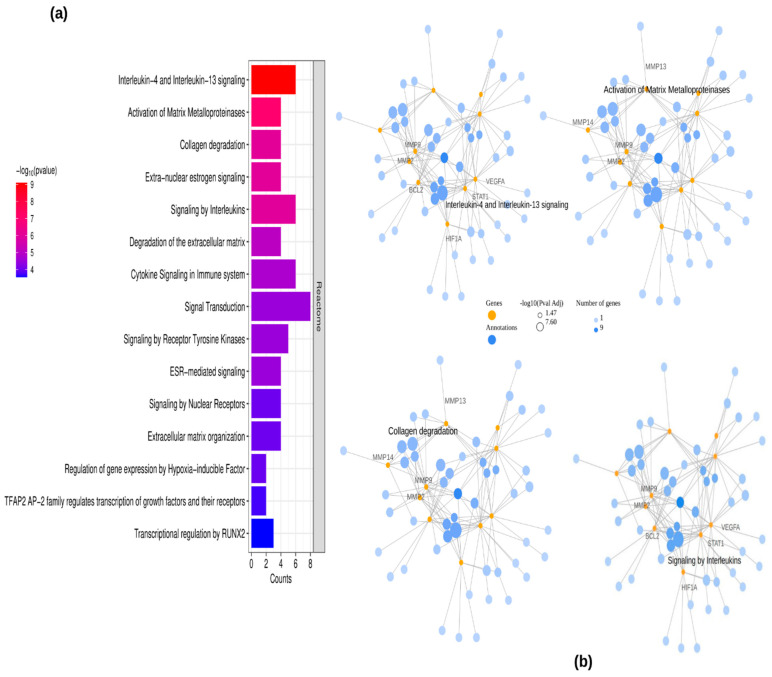

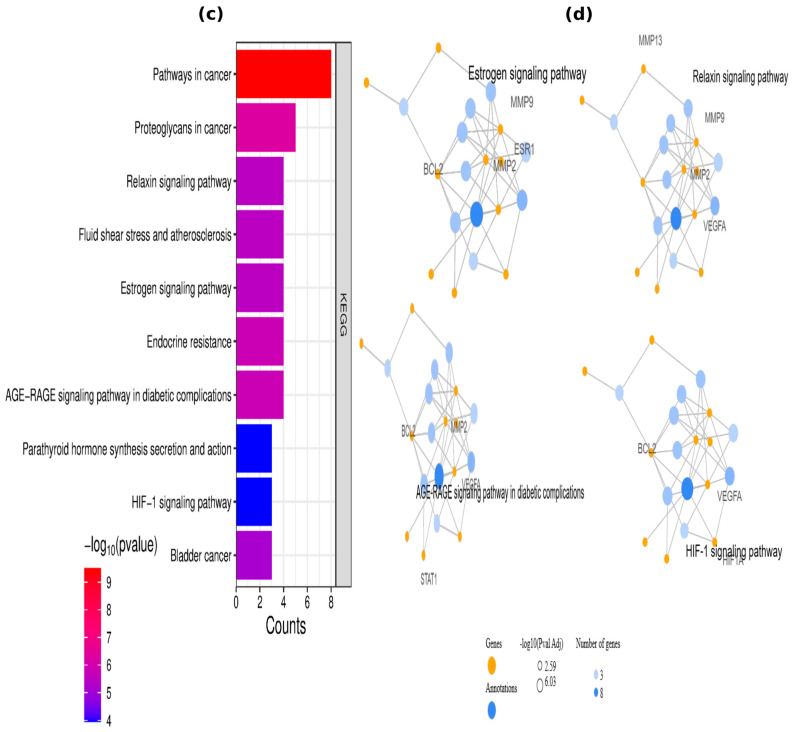
Results of REACTOME and KEGG pathway enrichment analysis and construction of the key pathway–target network. (**a**) Bar chart displaying the top 15 significantly enriched pathways based on REACTOME analysis. (**b**) Representative networks highlighting four key pathway terms from REACTOME, and their associated target genes. (**c**) Bar chart displaying the top 10 significantly enriched pathways based on KEGG analysis. (**d**) Representative networks highlighting four key pathway terms from KEGG, and their associated target genes. Color gradients represent varying *p* value thresholds, while bar lengths indicate the number of genes mapped to each pathway. Gene symbols—MMP2, MMP14, BCL2, STAT1, HIF1A, MMP9, MMP13, VEGFA, ESR1 and PPARG.

The REACTOME pathway analysis was conducted to investigate the functional roles and signaling mechanisms of the identified periodontitis-related targets of EGCG. A total of 139 significant pathways associated with periodontitis and EGCG were identified. Among these, the top 15 pathways with the highest gene counts were visualized in a bar plot (Figure 3a). The findings suggest that EGCG exerts its therapeutic effects on periodontitis through multiple targets and diverse signaling pathways. Furthermore, a network representation of key enriched pathways, including Interleukin-4 and Interleukin-13 signaling, Matrix Metalloproteinase activity, Collagen degradation, and Interleukin signaling, along with their essential target genes, is illustrated in Figure 3b. 

**Figure 4:**
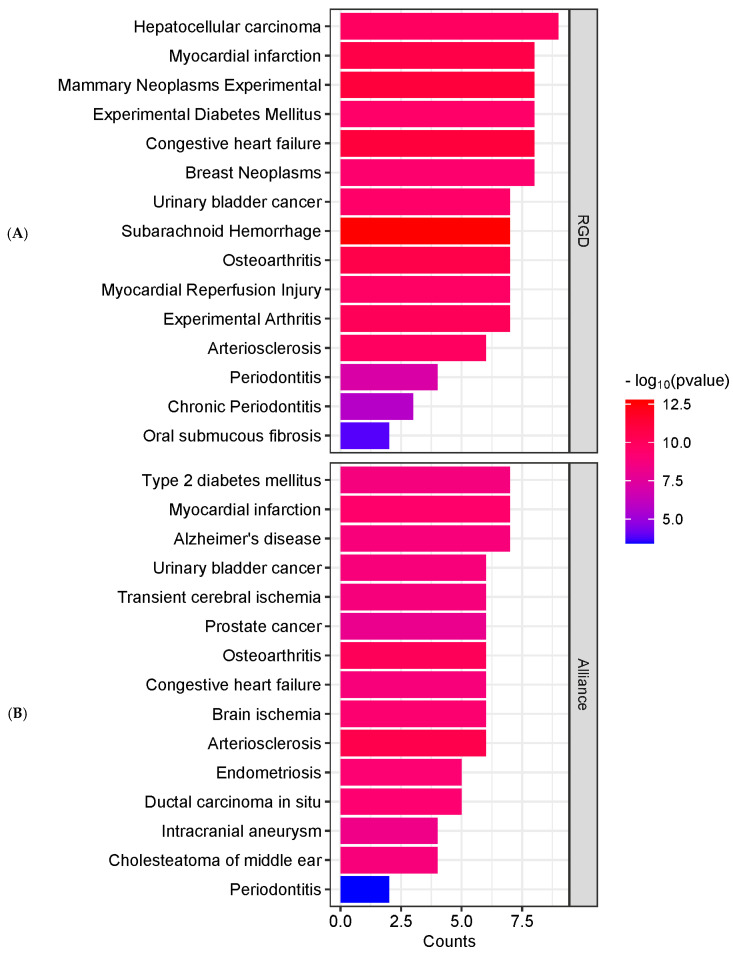
Results of disease enrichment analysis. (**A**) Bar chart showing the top 15 significantly enriched disease terms derived from the RGD disease database. (**B**) Bar chart showing the top 15 significantly enriched disease terms identified from the Alliance disease database. Color gradients indicate different *p* value thresholds, and bar lengths represent the number of genes associated with each disease term.

In addition to REACTOME analysis, KEGG pathway enrichment analysis was conducted to further explore the molecular mechanisms of EGCG against periodontitis. Several significantly enriched pathways were identified (Figure 3c), with the most prominent being pathways in cancer, proteoglycans in cancer, relaxin signaling, fluid shear stress and atherosclerosis, estrogen signaling, endocrine resistance, AGE–RAGE signaling in diabetic complications, parathyroid hormone signaling, and HIF-1 signaling. A network representation of these enriched pathways (Figure 3d) mapped key target genes, including MMP2, MMP9, MMP13, VEGFA, ESR1, STAT1, BCL2, and HIF1A, to the estrogen signaling, relaxin signaling, AGE–RAGE signaling, and HIF-1 signaling pathways. Additionally, disease enrichment analysis was performed using the RGD and Alliance databases to identify disease associations (Figure 4A,B). The results demonstrated that periodontitis and chronic periodontitis were significantly enriched, as reflected by their *p* values in both RGD and Alliance disease enrichment analyses.

### 2.4. Construction and Analysis of PPI Networks

**Figure 5:**
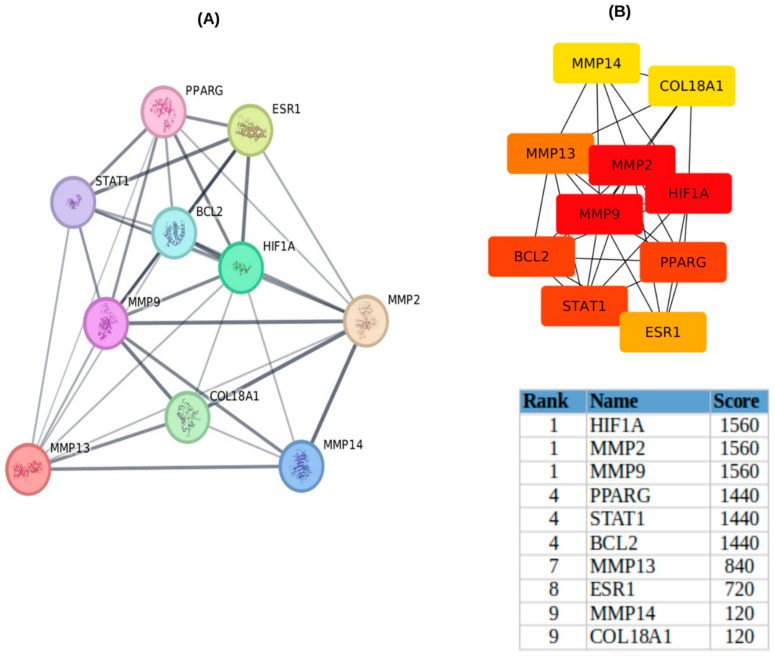
Protein–protein interaction (PPI) network and Cytoscape visualization of shared target genes between EGCG and periodontitis. (**A**) Protein–protein interaction (PPI) network constructed using the STRING database, based on the shared gene targets of EGCG and periodontitis. (**B**) Cytoscape analysis illustrating key target genes within the PPI network. Node color intensity follows an orange gradient representing degree centrality: darker shades indicate nodes with higher scores (i.e., greater connectivity to other nodes), whereas lighter shades represent nodes with lower connectivity. Gene symbols—MMP2, MMP14, BCL2, STAT1, HIF1A, MMP9, MMP13, COL18A1, ESR1 and PPARG.

The common genes identified between EGCG, and periodontitis were analyzed using the STRING database v11.0 to construct a PPI network and extract relevant protein-related data (Figure 5A). The resulting network consisted of 10 nodes and 36 edges, illustrating the interactions among the target genes. To enhance visualization, VEGFA was mapped to COL18A1 within the STRING database. The finalized network, displaying all 10 core target genes (MMP2, MMP14, BCL2, STAT1, HIF1A, MMP9, MMP13, COL18A1, ESR1, and PPARG), is presented in Figure 2a. For further exploration of protein–protein interactions, the STRING-derived PPI data were imported into Cytoscape 3.10.2, where a reconstructed PPI network containing 10 nodes and 36 edges was developed (Figure 5B). The CytoHubba plugin was utilized to identify key hub genes, with the MCC algorithm (score ≥ 720) highlighting the top eight hub genes: MMP2, BCL2, STAT1, HIF1A, MMP9, MMP13, ESR1, and PPARG (Figure 5B). By integrating these results with Gene Ontology, pathway, and disease enrichment analyses, it was determined that ESR1, MMP2, MMP9, MMP13, and STAT1 are potential key targets of EGCG in the context of periodontitis treatment. These five selected genes were subsequently used for docking and simulation analysis to assess their interactions with EGCG.

### 2.5. Molecular Docking

Molecular docking analysis was conducted using five candidate target proteins: ESR1 (PDB ID: 3ERT), MMP2 (PDB ID: 8H78), MMP9 (PDB ID: 1GKC), MMP13 (PDB ID: 2OW9), and STAT1 (PDB ID: 1BF5) to evaluate their interactions with EGCG. The 3D structure, molecular formula, and molecular weight of EGCG are provided in Table 1. The collected PDB structures of ESR1, MMP2, MMP9, MMP13, and STAT1 were obtained in complex with co-crystal ligands CCD, SPC, and Rutin, which served as reference structures for docking analysis.

**Figure 6:**
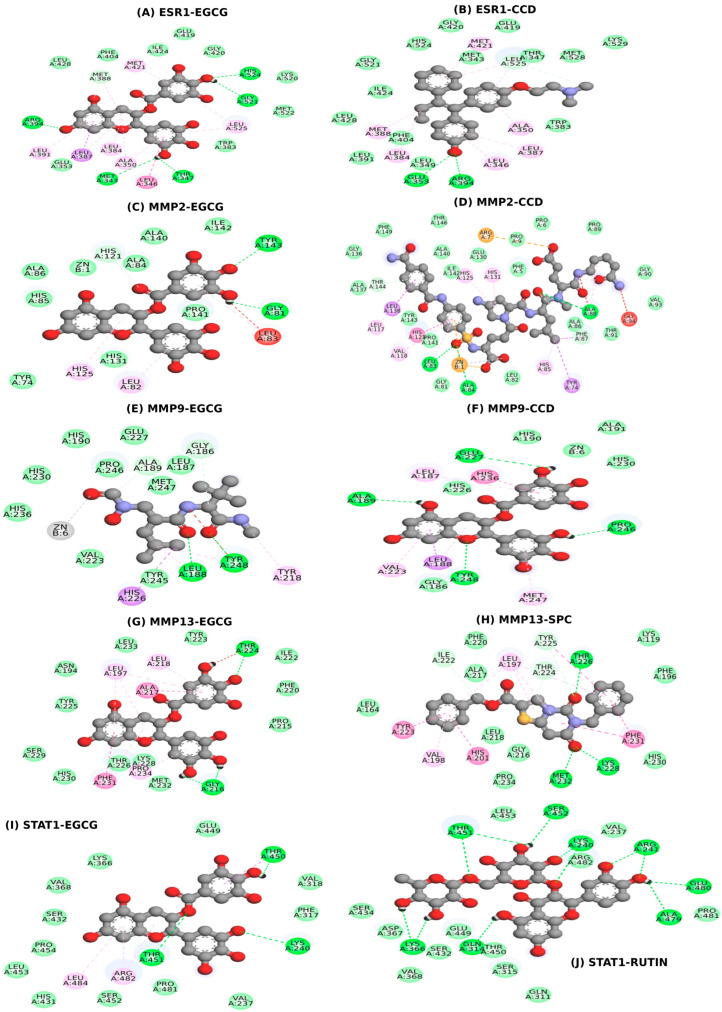
Molecular docking visualization of EGCG and reference compounds with key hub proteins involved in periodontitis. The docking complexes include: (**A**) ESR1–EGCG, (**B**) ESR1–CCD, (**C**) MMP2–EGCG, (**D**) MMP2–CCD, (**E**) MMP9–EGCG, (**F**) MMP9–CCD, (**G**) MMP13–EGCG, (**H**) MMP13–SPC, (**I**) STAT1–EGCG, and (**J**) STAT1–Rutin. The diagrams display ligand–protein interactions, highlighting the binding orientations within the active sites of each target. Gene symbols—ESR1, MMP2; MMP9; MMP13 and STAT1; EGCG, epigallocatechin gallate; CCD, co-crystal ligand; SPC, co-crystal ligand.

The docking results demonstrated strong binding interactions between EGCG and the selected target proteins. ESR1 interacted with both EGCG and CCD, with docking scores of −8.2 and −9.5, respectively, as illustrated in Figure 6A–B, with corresponding interactive residues listed in Table 2. Similarly, MMP2 exhibited binding with EGCG and CCD, with docking scores of −7.4 and −9.9, respectively, as shown in Figure 6C–D. For MMP9, the docking analysis revealed interactions with EGCG and CCD, with scores of −8.3 and −6.7, respectively, as visualized in Figure 6E–F. The protein MMP13 displayed strong binding affinity with EGCG and SPC, with docking scores of −8.8 and −12.3, respectively, as depicted in Figure 6G–H. Additionally, STAT1 interacted with EGCG and Rutin, yielding docking scores of −7.3 and −9.0, respectively, as shown in Figure 6I–J. The interactive residues for each docking pair are summarized in Table 2.

### 2.6. MD Simulations

To evaluate the stability of protein–ligand interactions and the structural flexibility of the proteins ESR1, MMP2, MMP9, MMP13, and STAT1 within their respective docked complexes with EGCG, further Molecular Dynamics (MD) simulations were conducted using GROMACS for 300 ns. In the present study, the MD simulations aimed to capture the dynamic changes that occur when the target proteins bind to the ligand. Several key parameters, including RMSD, RMSF, Rg, SASA, hydrogen bonding interactions, and PCA were computed to assess both the protein and protein–ligand complexes’ stability and dynamics throughout the simulation. The average value of apo and complex form of ESR1, MMP2, MMP9, MMP13, and STAT1 are shown in Table 3.

#### 2.6.1. ESR1 and ESR1–EGCG

**Figure 7:**
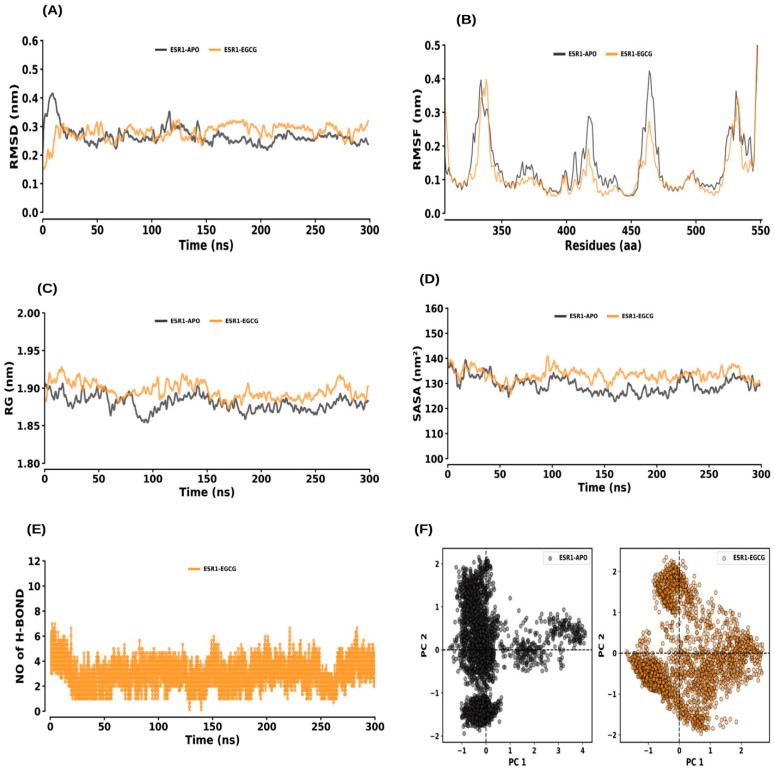
Molecular dynamics (MD) simulation analysis of ESR1 and its interaction with EGCG. (**A**) RMSD plot comparing the ESR1 apo form and ESR1–EGCG complex. (**B**) RMSF plot showing the residue-wise flexibility for ESR1 apo and ESR1–EGCG complex. (**C**) Rg distribution plot comparing the compactness of ESR1 in its apo form and the ESR1–EGCG complex. (**D**) SASA plot comparing the solvent-accessible surface area of ESR1 apo and ESR1–EGCG complex. (**E**) Time evolution of intermolecular hydrogen bonds formed in the ESR1–EGCG complex. (**F**) PCA plot illustrating the conformational dynamics of ESR1 in its apo form and in complex with EGCG.

The RMSD analysis demonstrated that both the ESR1–APO and ESR1–EGCG systems reached equilibrium within 200 ns and remained stable throughout the 300 ns simulation (Figure 7). The average RMSD values for ESR1–APO and ESR1–EGCG were 0.27 ± 0.03 nm and 0.28 ± 0.03 nm, respectively, suggesting minimal deviations during the simulation (Figure 7A). The RMSF analysis of the protein residues showed average values for ESR1–APO and ESR1–EGCG of 0.15 ± 0.12 nm and 0.13 ± 0.14 nm, respectively, indicating no significant change in the flexibility of the complexes with ligand binding (Figure 7B). The calculated Rg values were 1.88 ± 0.01 nm for ESR1–APO and 1.90 ± 0.01 nm for ESR1–EGCG, indicating negligible effects of EGCG binding on the protein’s compactness (Figure 7C). SASA values were 129.45 ± 3.66 nm^2^ for ESR1–APO and 133.32 ± 2.91 nm^2^ for ESR1–EGCG, suggesting a slight increase in solvent accessibility upon ligand binding (Figure 7D). Hydrogen bond formation between ESR1 and EGCG was consistent throughout the simulation, maintained by the average rate of 1 to 4 hydrogen bonds with the ESR1–EGCG complex. (Figure 7E). PCA of ESR1–APO and ESR1–EGCG showed average co-variance values of 26.63 nm and 26.77 nm, respectively (Figure 7F).

#### 2.6.2. MMP2 and MMP2–EGCG

**Figure 8:**
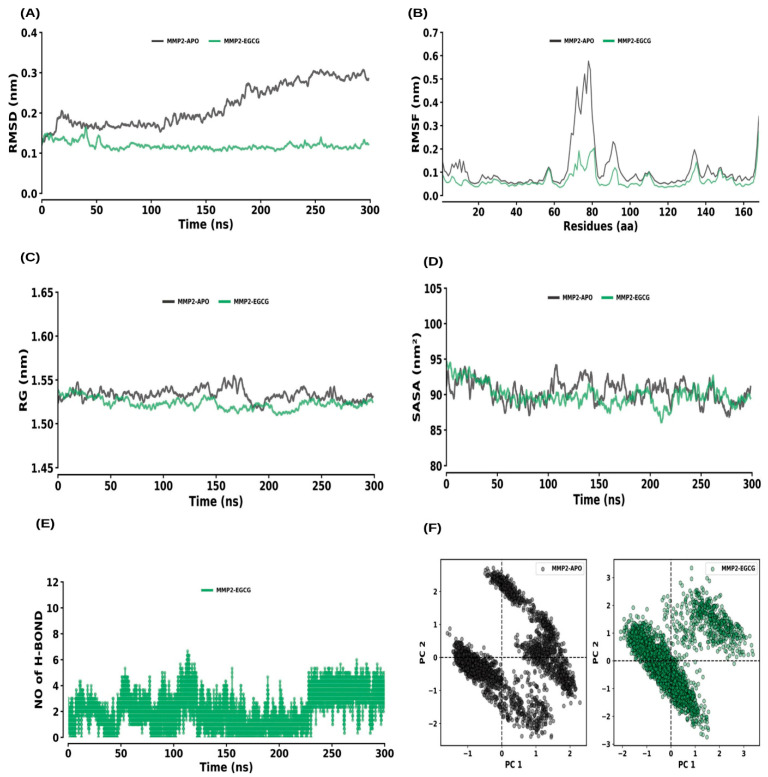
Molecular dynamics (MD) simulation analysis of MMP2 and its interaction with EGCG. (**A**) RMSD plot comparing the MMP2 apo form and MMP2–EGCG complex. (**B**) RMSF plot showing the residue-wise flexibility for MMP2 apo and MMP2–EGCG complex. (**C**) Rg distribution plot comparing the compactness of MMP2 in its apo form and the MMP2–EGCG complex. (**D**) SASA plot comparing the solvent-accessible surface area of MMP2 apo and MMP2–EGCG complex. (**E**) Time evolution of intermolecular hydrogen bonds formed in the MMP2–EGCG complex. (**F**) PCA plot illustrating the conformational dynamics of MMP2 in its apo form and in complex with EGCG.

RMSD analysis indicated that both MMP2–APO and MMP2–EGCG reached equilibrium within 200 ns and exhibited stability throughout the simulation (Figure 8A). The average RMSD values for MMP2–APO and MMP2–EGCG were 0.22 ± 0.05 nm and 0.12 ± 0.01 nm, respectively. The RMSF values for MMP2–APO and MMP2–EGCG were 0.11 ± 0.10 nm and 0.07 ± 0.04 nm, respectively, showing no significant change in flexibility due to ligand binding (Figure 8B). Rg values were 1.53 ± 0.01 nm for MMP2–APO and 1.52 ± 0.01 nm for MMP2–EGCG, with negligible effect on the protein’s compactness (Figure 8C). SASA values were 90.43 ± 2.01 nm^2^ for MMP2–APO and 89.96 ± 1.93 nm^2^ for MMP2–EGCG, indicating a slight change in solvent accessibility (Figure 8D). Hydrogen bonds formed between MMP2 and EGCG were consistent, maintained by the average rate of 1 to 2 hydrogen bonds with the MMP2–EGCG complex (Figure 8E). PCA for MMP2–APO and MMP2–EGCG showed average co-variance values of 10.60 nm and 2.73 nm, respectively (Figure 8F).

#### 2.6.3. MMP9 and MMP9–EGCG

**Figure 9:**
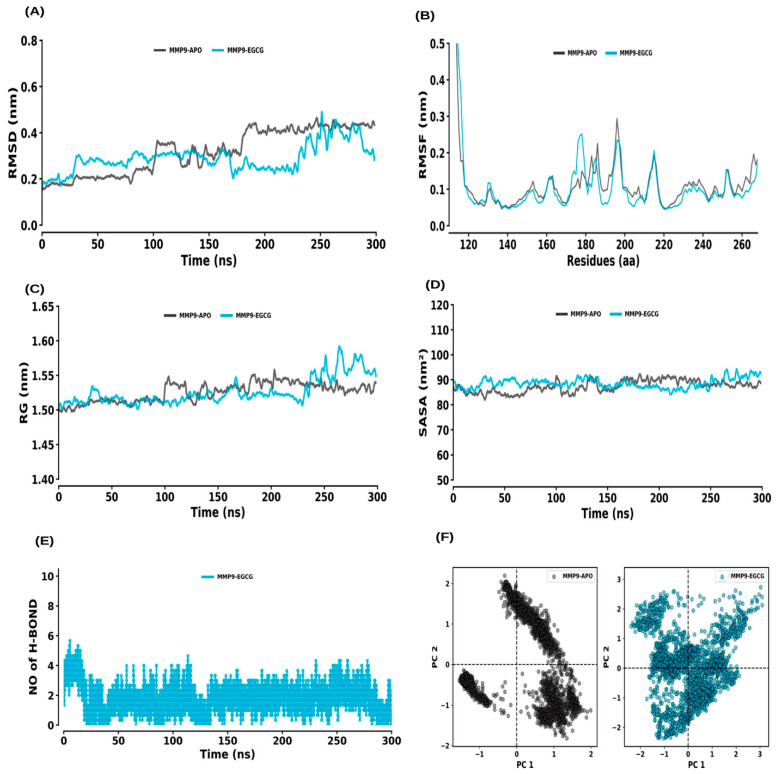
Molecular dynamics (MD) simulation analysis of MMP9 and its interaction with EGCG. (**A**) RMSD plot comparing the MMP9 apo form and MMP9–EGCG complex. (**B**) RMSF plot showing the residue-wise flexibility for MMP9 apo and MMP9–EGCG complex. (**C**) Rg distribution plot comparing the compactness of MMP9 in its apo form and the MMP9–EGCG complex. (**D**) SASA plot comparing the solvent-accessible surface area of MMP9 apo and MMP9–EGCG complex. (**E**) Time evolution of intermolecular hydrogen bonds formed in the MMP9–EGCG complex. (**F**) PCA plot illustrating the conformational dynamics of MMP9 in its apo form and in complex with EGCG.

The RMSD analysis for MMP9–APO and MMP9–EGCG showed equilibrium reached within 200 ns and stable systems through the 300 ns simulation (Figure 9A). The average RMSD values for MMP9–APO and MMP9–EGCG were 0.32 ± 0.10 nm and 0.29 ± 0.06 nm, respectively. RMSF values for MMP9–APO and MMP9–EGCG were 0.13 ± 0.17 nm and 0.12 ± 0.13 nm, respectively, with no significant change in flexibility (Figure 9B). The Rg values were 1.53 ± 0.01 nm for MMP9–APO and 1.53 ± 0.02 nm for MMP9–EGCG, showing no impact on compactness (Figure 9C). SASA values were 87.39 ± 2.70 nm^2^ for MMP9–APO and 88.58 ± 2.40 nm^2^ for MMP9–EGCG, indicating slight changes in solvent accessibility (Figure 9D). Hydrogen bond interactions were stable throughout the simulation and maintained by the average rate of 1 to 3 hydrogen bonds with the MMP9–EGCG complex (Figure 9E). PCA showed average co-variance values of 21.25 nm and 26.12 nm for MMP9–APO and MMP9–EGCG, respectively (Figure 9F).

#### 2.6.4. MMP13 and MMP13–EGCG

**Figure 10:**
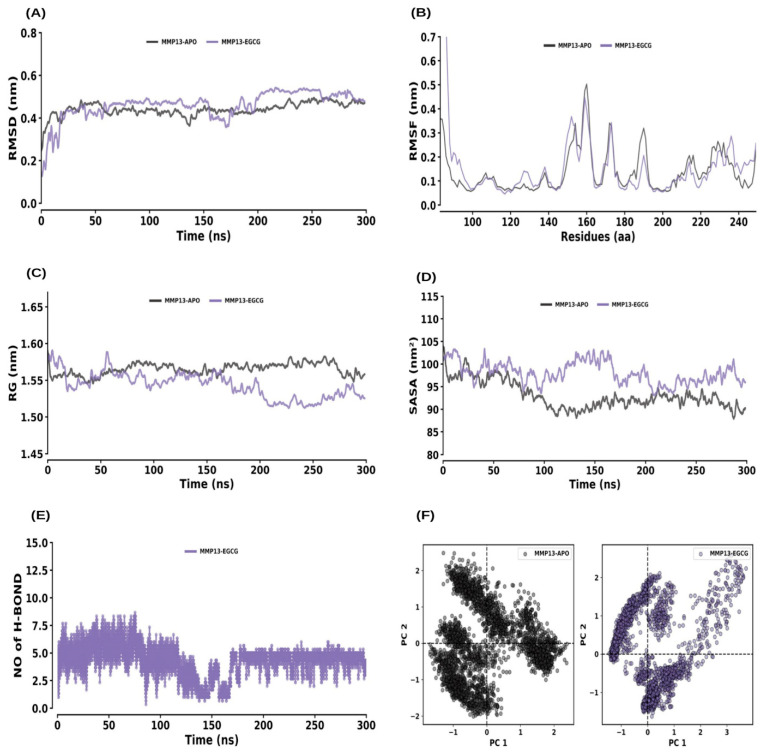
Molecular dynamics (MD) simulation analysis of MMP13 and its interaction with EGCG. (**A**) RMSD plot comparing the MMP13 apo form and MMP13–EGCG complex. (**B**) RMSF plot showing the residue-wise flexibility for MMP13 apo and MMP13–EGCG complex. (**C**) Rg distribution plot comparing the compactness of MMP13 in its apo form and the MMP13–EGCG complex. (**D**) SASA plot comparing the solvent-accessible surface area of MMP13 apo and MMP13–EGCG complex. (**E**) Time evolution of intermolecular hydrogen bonds formed in the MMP13–EGCG complex. (**F**) PCA plot illustrating the conformational dynamics of MMP13 in its apo form and in complex with EGCG.

For MMP13–APO and MMP13–EGCG, the RMSD analysis revealed equilibrium within 200 ns, with both systems maintaining stability through 300 ns (Figure 10A). The average RMSD values for MMP13–APO and MMP13–EGCG were 0.44 ± 0.03 nm and 0.46 ± 0.07 nm, respectively. RMSF values for MMP13–APO and MMP13–EGCG were 0.14 ± 0.09 nm and 0.16 ± 0.17 nm, respectively (Figure 10B). The Rg values were 1.57 ± 0.01 nm for MMP13–APO and 1.54 ± 0.02 nm for MMP13–EGCG, indicating negligible changes in compactness (Figure 10C). SASA values were 93.05 ± 3.32 nm^2^ for MMP13–APO and 98.05 ± 2.79 nm^2^ for MMP13–EGCG, reflecting a slight increase in solvent accessibility (Figure 10D). Consistent hydrogen bond formation was observed throughout the simulation and maintained by the average rate of 1 to 5 hydrogen bonds with the MMP13–EGCG complex (Figure 10E). PCA showed average co-variance values of 12.84 nm and 14.02 nm for MMP13–APO and MMP13–EGCG, respectively (Figure 10F).

#### 2.6.5. STAT1 and STAT1–EGCG

**Figure 11:**
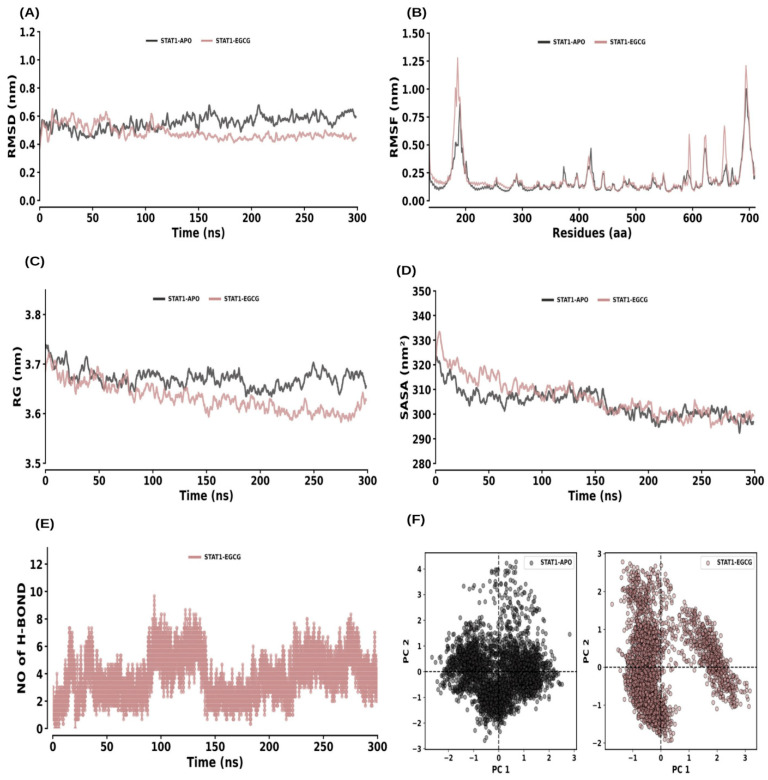
Molecular dynamics (MD) simulation analysis of STAT1 and its interaction with EGCG. (**A**) RMSD plot comparing the STAT1 apo form and STAT1–EGCG complex. (**B**) RMSF plot showing the residue-wise flexibility for STAT1 apo and STAT1–EGCG complex. (**C**) Rg distribution plot comparing the compactness of STAT1 in its apo form and the STAT1–EGCG complex. (**D**) SASA plot comparing the solvent-accessible surface area of STAT1 apo and STAT1–EGCG complex. (**E**) Time evolution of intermolecular hydrogen bonds formed in the STAT1–EGCG complex. (**F**) PCA plot illustrating the conformational dynamics of STAT1 in its apo form and in complex with EGCG.

RMSD analysis showed that both STAT1–APO and STAT1–EGCG reached equilibrium within 200 ns and remained stable throughout the 300 ns simulation (Figure 11A). The average RMSD values for STAT1–APO and STAT1–EGCG were 0.55 ± 0.06 nm and 0.48 ± 0.06 nm, respectively. The RMSF analysis for STAT1–APO and STAT1–EGCG showed average values of 0.18 ± 0.13 nm and 0.21 ± 0.18 nm, respectively (Figure 11B). The Rg values were 3.67 ± 0.02 nm for STAT1–APO and 3.63 ± 0.03 nm for STAT1–EGCG, indicating no significant change in compactness (Figure 11C). SASA values were 304.35 ± 5.97 nm^2^ for STAT1–APO and 306.82 ± 8.18 nm^2^ for STAT1–EGCG, showing a slight increase in solvent accessibility (Figure 11D). Hydrogen bonds were consistently formed throughout the simulation and maintained by the average rate of 1 to 3 hydrogen bonds with the STAT1–EGCG complex (Figure 11E). The PCA showed average co-variance values of 83.65 nm and 125.44 nm for STAT1–APO and STAT1–EGCG, respectively (Figure 11F).

### 2.7. MM–PBSA Calculation

To further evaluate the binding energy of ESR1–EGCG, MMP2–EGCG, MMP9–EGCG, MMP13–EGCG and STAT1–EGCG complexes we performed MM–PBSA calculations. The MM–PBSA binding energy scoring function was applied by calculating a weighted sum of multiple energy components, including van der Waals (vdW), electrostatic (Elec), polar solvation, and solvent-accessible surface area (SASA). The binding score of ESR1–EGCG, MMP2–EGCG, MMP9–EGCG, MMP13–EGCG and STAT1–EGCG complexes were −113.37 ± 10.75, −31.63 ± 9.74, −77.48 ± 21.45, −101.94 ± 14.42 and −28.04 ± 13.04, respectively, shown in Table 4.

## 3. Discussion

The findings of this study provide significant insights into the potential molecular mechanisms underlying the therapeutic effects of epigallocatechin-3-gallate (EGCG) in periodontitis. By integrating target identification, enrichment analysis, PPI network analysis, molecular docking, and molecular dynamics (MD) simulations, this study identifies key genes and pathways that may mediate the beneficial effects of EGCG in periodontitis treatment.

The identification of 14 potential EGCG-associated targets and 527 periodontitis-related targets led to the discovery of 10 shared genes, including MMP2, MMP14, BCL2, STAT1, HIF1A, MMP9, MMP13, VEGFA, ESR1, and PPARG (Figure 1). These genes are involved in various biological processes relevant to periodontitis, such as inflammation, extracellular matrix remodeling, and tissue repair. Enrichment analysis further confirmed the functional relevance of these genes by highlighting their involvement in biological processes like response to hypoxia, collagen metabolism, and epithelial morphogenesis. The extracellular matrix and zinc ion binding functions were also found to be significantly enriched, reinforcing the importance of these targets in periodontal pathophysiology (Figure 2a–d).

Notably, GO enrichment profiling revealed that these target genes participate in biological processes such as tissue development and ossification, which are disrupted during periodontitis and responsible for bone loss and defective repair. Response to hypoxia, mediated by HIF1A and VEGFA, shapes the inflammatory microenvironment and impairs healing in periodontal lesions [27]. Collagen metabolic pathways governed by MMPs are pivotal for attachment loss and tissue destruction [28]. Within the cellular component category, the extracellular matrix and transcription complexes are essential for matrix turnover and gene regulation; MMP2, MMP9, MMP13, and MMP14 are central players here [16,17]. Zinc ion binding and metallopeptidase activities molecular functions tightly associated with these MMPs underscore their destructive capability, while the nuclear receptor ESR1 also modulates inflammation and bone homeostasis [28]. EGCG interacts with these proteins, acting as a potent inhibitor of MMPs and modulator of immune signaling, thereby mitigating matrix degradation and inflammation [28,29]. These GO pathway connections help clarify the multifaceted biological actions of EGCG in the context of periodontitis.

The REACTOME pathway analysis revealed that EGCG may exert its effects through multiple biological pathways, including interleukin signaling, matrix metalloproteinase (MMP) activity, and collagen degradation, which are critical for tissue remodeling, inflammation, and immune response in periodontitis [16,30,31,32]. KEGG pathway enrichment further highlighted estrogen signaling, relaxin signaling, AGE–RAGE signaling in diabetic complications, and HIF-1 signaling [33,34,35] as key mechanisms targeted by EGCG, encompassing extracellular matrix remodeling, angiogenesis, immune modulation, and hypoxia-driven responses [36,37,38]. The consistent presence of MMP2, MMP9, and MMP13 in both enrichment and pathway network analyses (Figure 3a–d) underscores their pivotal role in ECM degradation and tissue destruction, suggesting that EGCG’s inhibitory interaction with these MMPs may contribute to its protective effects in periodontal tissues. Protein–protein interaction (PPI) network analysis identified ESR1, MMP2, MMP9, MMP13, and STAT1 as hub genes, highlighting their central role in EGCG-mediated modulation of periodontitis [14,15,16,17,18,30,31,32,33,34,35,36,37,38]. Notably, ESR1 is involved in bone metabolism and inflammatory regulation [14,15], while STAT1 is a key transcription factor in immune signaling [18], together supporting the multifunctional potential of EGCG to limit tissue breakdown and modulate immune responses (Figure 5).

Absorption, distribution, metabolism, excretion, and toxicity (ADMET) properties are critical for evaluating drug-likeness. Although EGCG demonstrated strong binding in docking and MD simulations, its pharmacokinetic profile presents challenges. Key physicochemical properties include a molecular weight of 458.4 g/mol, XLogP3 of 1.2, 8 hydrogen bond donors, 11 hydrogen bond acceptors, and 4 rotatable bonds. These parameters result in two Lipinski’s rule violations (HBD > 5; HBA > 10), indicating limited oral drug-likeness. These findings are summarized in Table S3, highlighting EGCG’s restricted systemic availability and dose-dependent safety considerations that should be accounted for when interpreting network pharmacology predictions.

Molecular docking analysis demonstrated strong binding affinities between EGCG and the selected target proteins, with docking scores indicating favorable interactions (Figure 6A–J). ESR1, MMP2, MMP9, MMP13, and STAT1 exhibited docking scores ranging from −7.3 to −8.8 (Table 2), suggesting stable ligand–receptor complexes. Notably, hydrogen bonding interactions were observed between EGCG and these proteins, which likely contribute to the stability and specificity of binding. These findings are consistent with previous reports highlighting hydrogen bonding as a key factor in the binding affinity and biological activity of polyphenolic compounds [39,40]. The strong interactions of EGCG with ESR1, MMP2, MMP9, MMP13, and STAT1 support its potential to modulate MMP activity and reduce extracellular matrix degradation, thereby mitigating tissue destruction in periodontitis.

To further validate these molecular interactions, MD simulations were performed for 300 ns to assess the stability and dynamic behavior of the EGCG–protein complexes (Figure 7, Figure 8, Figure 9, Figure 10 and Figure 11). The results demonstrated that all complexes remained stable throughout the simulation, with minimal fluctuations in RMSD, RMSF, and Rg values (Table 3). The consistent hydrogen bonding interactions between EGCG and the target proteins reinforced the stability of these interactions. Interestingly, the binding of EGCG to ESR1, MMP9, and MMP13 did not significantly alter the structural compactness of these proteins, suggesting that EGCG binding does not induce major conformational changes but may still modulate their enzymatic activity. The MM–PBSA calculations further provided quantitative insights into the binding free energy of the EGCG–protein complexes. The ESR1–EGCG, and MMP13–EGCG complexes exhibited the highest binding affinities, with binding scores of −113.37 and −101.94, respectively, indicating strong and stable interactions (Table 4). MMP2, MMP9 and STAT1 also showed favorable binding energies, suggesting that EGCG effectively interacts with these proteins to regulate their functions. The high binding affinity of EGCG for ESR1 is particularly noteworthy, as ESR1 plays a role in bone homeostasis and inflammation, both of which are critical in periodontitis progression.

These computational and simulation findings regarding EGCG’s multi-target molecular interactions are strongly supported by experimental results. For instance, Cai et al. demonstrated that green tea epigallocatechin-3-gallate significantly alleviates Porphyromonas gingivalis-induced periodontitis in mice, reducing inflammation and tissue destruction [41]. This in vivo evidence validates the predicted inhibitory effects of EGCG on key targets such as MMPs and inflammatory regulators, reinforcing its therapeutic potential in periodontal disease.

Overall, these findings provide compelling evidence that EGCG interacts with key molecular targets involved in periodontitis, particularly MMPs and inflammatory regulators. By modulating these targets, EGCG may help mitigate tissue destruction and inflammation associated with periodontal disease. The identification of ESR1 as a potential therapeutic target further highlights the possible involvement of estrogen receptor signaling in the pathogenesis of periodontitis and suggests that EGCG may have broader implications beyond its antioxidant and anti-inflammatory properties. These results align with previous studies that have demonstrated the anti-inflammatory and MMP-inhibitory effects of EGCG in periodontal disease models. However, this study advances the understanding of EGCG’s molecular mechanisms by providing a comprehensive analysis of its potential targets, pathways, and interactions. Future experimental studies, including in vitro and in vivo validations, are warranted to confirm these computational findings and further explore the therapeutic potential of EGCG in clinical settings.

The study does have some limitations, primarily related to the computational nature of the analysis. While molecular docking and MD simulations provide strong theoretical support for EGCG’s interactions with periodontitis-related targets, experimental validation is essential to confirm these interactions in a biological context. Additionally, factors such as bioavailability, metabolic stability, and dosage optimization need to be considered in future studies to determine the clinical relevance of EGCG in periodontal therapy.

In summary, this study provides a comprehensive computational framework for understanding the molecular mechanisms of EGCG in periodontitis treatment. By identifying key gene targets, enriched pathways, and stable molecular interactions, the findings support the therapeutic potential of EGCG in modulating key molecular mechanisms involved in periodontal disease. These insights pave the way for further experimental research to validate EGCG as a promising natural compound for periodontitis treatment.

## 4. Materials and Methods

### 4.1. Epigallocatechin-3-gallate (EGCG) Compound Dataset and Target Prediction

EGCG target genes were collected from two databases: The SwissTarget Prediction database [42] was used for the prediction of small molecule targets by calculating the EGCG molecule structural formula and target screening using probability > 0 and origin as human. In the TCMSP Database [43], corresponding targets were collected by using the “epigallocatechin-3-gallate” keyword search. Target names were amended by the UNIPROT database (gene source: human) [44].

### 4.2. Periodontitis Target Prediction

Periodontitis target genes were retrieved from two databases: GeneCards V3.0 database [45], and GEO database [46]. The targets of the disease associated with periodontitis were predicted by entering the keyword “Periodontitis”, and targets from the GeneCards database with a relevance score > 2 were screened and the results from each database were collated and de-weighted to derive periodontitis-related targets. Target names were modified by the UNIPROT database (gene source: human) [44].

### 4.3. The Intersection of EGCG and Periodontitis Targets and Construction of PPI Networks

The target information obtained was used to map the Venn diagram of periodontitis and EGCG with the online software Jvenn online software [47], in order to determine the common targets. The target genes that were acquired were imported into the STRING V12.0 platform [48] in order to analyze the protein–protein interaction (PPI) using the screening condition of “Homo sapiens” and a confidence level of 0.9. To identify regions with high interconnectivity within the PPI network, we employed the Cytoscape V3.10.2 plugin cytoHubba [49,50]. The MCC algorithm was employed to analyze the node degree of key nodes within significant modules, enabling the identification of hub genes within the PPI network.

### 4.4. Enrichment Analysis

To conduct enrichment analysis using R Shiny GO v0.81 [51], hub genes identified through a network pharmacology approach are uploaded to the platform. After selecting the appropriate species, the input gene list, formatted as gene symbols, is processed for functional enrichment. The tool associates these genes with biological categories, including Gene Ontology (GO) terms, REACTOME pathways, KEGG pathways and disease-related enrichments. Statistical methods, such as the hypergeometric test, are employed to identify categories that are significantly enriched in the gene set compared to the background. Results are filtered based on a false discovery rate (FDR) threshold and ranked by fold enrichment. The most significant terms, including the top 10 GO terms and top 15 REACTOME, disease enrichment and Drug enrichment categories, are visualized using SRplot [52]. SRplot effectively summarizes the significance and biological relevance of enriched terms, offering a clear, graphical representation of the results. These visualizations and outputs provide valuable insights and aid in the interpretation and communication of meaningful biological findings.

### 4.5. Docking

Molecular docking of Epigallocatechin gallate with key targets associated with periodontitis, including ESR1, MMP2, MMP9, MMP13, and STAT1, was performed using AutoDock Vina v1.1.2 [53]. The 3D structure of Epigallocatechin-3-gallate (EGCG) was downloaded from the PubChem database [54] and converted from SDF to PDBQT format using Open Babel 3.1.1 [55]. Ligand energy minimization was performed in Avogadro v1.2.0 [56] employing the MMFF94 force field with the Steepest Descent algorithm for 5000 steps, updating the conformation at each step until the change in energy was less than 0.1. Although ab initio quantum chemical calculations can offer higher precision in atomic positioning, MMFF94-based minimization was used here to maintain a balance between computational efficiency and accuracy. This approach is widely utilized in molecular docking studies and provides reliable ligand geometries, with subsequent molecular dynamics simulations further refining the interactions between EGCG and target proteins while accounting for their structural flexibility. The minimized ligand was saved in PDB format for use in docking studies. The 3D crystallographic structures of ESR1 (3ERT), MMP13 (2OW9), MMP2 (8H78), MMP9 (1GKC), and STAT1 (1BF5) were obtained from the RCSB–PDB database. Water molecules were removed using mgltools_x86_64Linux2_1.5.7, and the protein structures were prepared by adding hydrogen atoms and assigning Kollman charges before conversion to pdbqt format via Open Babel 3.1.0. Molecular docking in AutoDock Vina was performed with consistent parameters across all targets, except for grid center positions. Grid maps were prepared using AutoGrid4, and grid box dimensions were set as ESR1 (X = 16, Y = 12, Z = 16), MMP13 (X = 16, Y = 10, Z = 16), MMP2 (X = 22, Y = 28, Z = 18), MMP9 (X = 10, Y = 10, Z = 14), and STAT1 (X = 20, Y = 28, Z = 18), maintaining a grid spacing of 1 Å. Grid centers were defined as ESR1 (30.038, −1.527, 24.784), MMP13 (52.617, 9.913, 11.497), MMP2 (27.753, 24.753, −8.468), MMP9 (65.157, 31.349, 116.472), and STAT1 (64.915, 45.125, 84.501), covering binding sites for ligand movement and rotation. Docked conformations were ranked by binding energy, and interactions were analyzed using PyMOL 3.1 [57] and Discovery Studio Visualizer v2.0 [58].

### 4.6. MD Simulation and MM_PBSA

The computational workflow for each protein–ligand complex was carried out systematically, following several essential steps. Initially, the docked complexes, including ESR1–EGCG, MMP2–EGCG, MMP9–EGCG, MMP13–EGCG, and STAT1–EGCG, were chosen for further investigation. The ligand topology was generated using the ATB server [59], which performs ab initio structural optimization with the 6-31G* basis set, ensuring quantum chemically accurate ligand geometries for MD simulations. Hydrogen atoms were added to the heavy atoms of each complex using the pdb2gmx module in GROMACS-2019.4 [60]. Subsequently, vacuum minimization was performed for 1500 steps utilizing the steepest descent algorithm. Following minimization, the structures were solvated within a cubic periodic box using the simple point charge (SPCE) water model. To maintain a salt concentration of 0.15 M, Na and Cl counter ions were introduced. System preparation followed a previously established protocol [61]. After system equilibration under NPT conditions, 300 ns production runs were conducted for each complex to assess structural stability and dynamic behavior. Simulation trajectories were analyzed using various GROMACS tools [60] to assess structural and dynamic properties, including root mean square deviation (RMSD), root mean square fluctuation (RMSF), radius of gyration (RG), solvent-accessible surface area (SASA), hydrogen bonding (H-Bond), and principal component analysis (PCA). Binding free energies of the complexes were calculated using the MM–PBSA approach via the g_mmpbsa tool [62], based on the last 50 ns of the trajectories with a timestep of 1000 frames. This approach provides detailed insights into protein–ligand interactions, with long simulation times ensuring equilibration and stability, even though independent replicates were not performed.

## 5. Conclusions

This study demonstrates the therapeutic potential of epigallocatechin-3-gallate (EGCG) in periodontitis by identifying and targeting key proteins involved in inflammation and tissue degradation. Computational analyses revealed strong and stable interactions between EGCG and ESR1, MMP2, MMP9, MMP13, and STAT1. Notably, EGCG showed the highest binding affinities with ESR1 (−113.37 ± 10.75 K/Cal) and MMP13 (−101.94 ± 14.42 K/Cal), suggesting effective inhibition of matrix metalloproteinases and modulation of extracellular matrix remodeling. Hydrogen bonding interactions further supported the stability of these complexes, while MM–PBSA calculations confirmed favorable binding energies and energetic stability. Although these results provide mechanistic insights, experimental validation is required to confirm EGCG’s biological activity. Future in vitro and in vivo studies should focus on verifying these protein–ligand interactions, evaluating bioavailability and metabolic stability, and optimizing dosage for clinical applications. Overall, these findings provide a solid computational foundation for considering EGCG as a promising natural therapeutic agent for managing periodontitis.

## Figures and Tables

**Table 1:** Basic information and chemical structure of epigallocatechin-3-gallate (EGCG).

Name	PubChem ID	MF	MW	Canonical Smiles	Structure
Epigallo catechin-3-gallate	65064	C22H18O11	458.4	C1[C@H]([C@H](OC2=CC(=CC(=C21)O)O)C3=CC(=C(C(=C3)O)O)O)OC(=O)C4=CC (=C(C(=C4)O)O)O	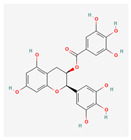

MF, molecular formula; MW, molecular weight.

**Table 2:** Binding affinities of EGCG and selected reference compounds with key hub proteins (ESR1, MMP2, MMP9, MMP13, and STAT1) associated with periodontitis, as determined by molecular docking analysis.

Target Name	Protein–Ligand Complex	Docking Score	Interactions
ESR1	ESR1–EGCG (Epigallocatechin gallate)	−8.2	MET343, LEU346, THR347, ALA350, GLU353, TRP383, LEU384, LEU387, MET388, LEU391, ARG394, PHE404, GLU419, GLY420, MET421, LYS520, GLY521, MET522, HIS524, LEU525,
	ESR1–CCD (Co-Crystal Ligand (Standard))	−9.5	MET343, LEU346, THR347, LEU349, ALA350, GLU353, TRP383, LEU384, LEU387, MET388, ARG394, PHE404, GLU419, GLY420, MET421, LEU428, GLY521, HIS524, LEU525, MET528,
MMP2	MMP2–EGCG (Epigallocatechin gallate)	−7.4	ZN1, TYR74, GLY81, LEU82, LEU83, ALA84, HIS85, ALA86, HIS121, HIS125, ALA140, PRO141, ILE142, TYR143,
	MMP2–CCD (Co-Crystal Ligand (Standard))	−9.9	ZN1, PHE5, PRO6, TYR74, GLY81, LEU82, LEU83, ALA84, HIS85, ALA86, PHE87, ALA88, PRO89, GLY90, THR91, VAL93, GLY94, LEU117, VAL118, HIS121, HIS125, GLU130, HIS131, GLY136, ALA137, LEU138, PRO141, ILE142, TYR143, THR144, PHE149,
MMP9	MMP9–EGCG (Epigallocatechin gallate)	−8.3	ZN6, GLY186, LEU187, LEU188, ALA189, ALA191, VAL223, HIS226, GLU227, HIS230, HIS236, PRO246, MET247, TYR248,
	MMP9–CCD (Co-Crystal Ligand (Standard))	−6.7	ZN6, GLY186, LEU187, LEU188, ALA189, TYR218, VAL223, HIS226, GLU227, HIS230, HIS236, TYR245, PRO246, MET247, TYR248,
MMP13	MMP13–EGCG (Epigallocatechin gallate)	−8.8	LEU197, PRO215, GLY216, ALA217, LEU218, ILE222, TYR223, THR224, TYR225, THR226, LYS228, SER229, HIS230, PHE231, MET232, PRO234,
	MMP13–SPC Co-Crystal Ligand (SPC) Standard	−12.3	LYS119, PHE196, LEU197, VAL198, HIS201, GLY216, ALA217, LEU218, PHE220, ILE222, TYR223, THR224, TYR225, THR226, LYS228, HIS230, PHE231, MET232, PRO234,
STAT1	STAT1–EGCG (Epigallocatechin gallate)	−7.3	LYS240, PHE317, LYS366, VAL368, HIS431, SER432, GLU449, THR450, THR451, SER452, LEU453, PRO454, ARG482, LEU484,
	STAT1–Rutin (Standard)	−9.0	VAL237, LYS240, ARG241, GLN314, SER315, LYS366, ASP367, SER432, SER434, GLU449, THR450, THR451, SER452, ALA479, GLU480, PRO481, ARG482,

Gene symbols—ESR1, MMP2; MMP9; MMP13, and STAT1; EGCG, epigallocatechin gallate; CCD, co-crystal ligand; SPC, co-crystal ligand.

**Table 3:** Average MDS values of RMSD, RMSF, Rg, SASA, NH-bonds, and covariance for hub proteins (ESR1, MMP2, MMP9, MMP13, and STAT1) in apo form and in complex with EGCG.

Protein Type	RMSD (nm)	RMSF (nm)	Rg (nm)	SASA (nm^2^)	NH-Bond (Protein–Drug)	Covariance
ESR1–APO ESR1–EGCG	0.27 ± 0.03	0.15 ± 0.12	1.88 ± 0.01	129.45 ± 3.66	NA	26.63
	0.28 ± 0.03	0.13 ± 0.14	1.90 ± 0.01	133.32 ± 2.91	4.0 ± 1.0	26.77
MMP2–APO MMP2–EGCG	0.22 ± 0.05	0.11 ± 0.10	1.53 ± 0.01	90.43 ± 2.01	NA	10.60
	0.12 ± 0.01	0.07 ± 0.04	1.52 ± 0.01	89.96 ± 1.93	3.0 ± 1.0	02.73
MMP9–APO MMP9–EGCG	0.32 ± 0.10	0.13 ± 0.17	1.53 ± 0.01	87.39 ± 2.70	NA	21.25
	0.29 ± 0.06	0.12 ± 0.13	1.53 ± 0.02	88.58 ± 2.40	2.0 ± 1.0	26.12
MMP13–APO MMP13–EGCG	0.44 ± 0.03	0.14 ± 0.09	1.57 ± 0.01	93.05 ± 3.32	NA	12.84
	0.46 ± 0.07	0.16 ± 0.17	1.54 ± 0.02	98.05 ± 2.79	5.0 ± 1.4	14.02
STAT1–APO STAT1–EGCG	0.55 ± 0.06	0.18 ± 0.13	3.67 ± 0.02	304.35 ± 5.97	NA	83.65
	0.48 ± 0.06	0.21 ± 0.18	3.63 ± 0.03	306.82 ± 8.18	3.5 ± 1.7	125.44

RMSD, root mean square deviation; RMSF, root mean square fluctuation; Rg, radius of gyration; SASA, solvent accessible surface area; NH-bond, number of hydrogen bonds.

**Table 4:** MM–PBSA binding free energy values for hub proteins (ESR1, MMP2, MMP9, MMP13, and STAT1) in complex with EGCG.

Complex Type	Binding Energy (K/Cal)	van der Waal Energy (K/Cal)	Electrostatic Energy (K/Cal)	Polar Solvation Energy (K/Cal)	SASA Energy (K/Cal)
ESR1–EGCG	−113.37 ± 10.75	−197.13 ± 12.46	−94.15 ± 17.39	199.88 ± 6.88	−21.97 ± 0.84
MMP2–EGCG	−31.63 ± 9.74	−101.99 ± 9.42	−14.67 ± 14.52	98.35 ± 22.39	−13.33 ± 0.95
MMP9–EGCG	−77.48 ± 21.45	−138.86 ± 11.84	−25.42 ± 37.36	103.03 ± 31.29	−16.23 ± 0.82
MMP13–EGCG	−101.94 ± 14.42	−217.54 ± 18.83	−147.19 ± 15.21	284.23 ± 13.69	−21.45 ± 0.74
STAT1–EGCG	−28.04 ± 13.04	−135.28 ± 17.71	−131.22 ± 34.87	256.03 ± 29.55	−17.56 ± 0.98

## Data Availability

All data generated or analyzed during this study are included in this published article.

## Supplementary Materials

The following supporting information can be downloaded at: https://www.mdpi.com/article/10.3390/ijms26189144/s1.
