# Supplementary material for: Exploring the Therapeutic Potential of Epigallocatechin-3-gallate (Green Tea) in Periodontitis Using Network Pharmacology and Molecular Modeling Approach
Source: Int J Mol Sci. 2025 Sep 19;26(18):9144. doi: 10.3390/ijms26189144 (PMC12470304; doi:10.3390/ijms26189144)
Supplement: Supplementary file 1 [file ijms-26-09144-s001.zip › Table S3.pdf]

Table S3: ADMET (Absorption, Distribution, Metabolism, Excretion, and Toxicity) profile of EGCG, including physicochemical properties, Lipinski's rule compliance, and implications for oral drug-likeness

Property	Value / Observation	Source / Note
Molecular weight (MW)	458.4 g/mol	PubChem
XLogP3 (lipophilicity)	1.2	PubChem
H-bond donors (HBD)	8	PubChem
H-bond acceptors (HBA)	11	PubChem
Rotatable bonds	4	PubChem
Lipinski's rule compliance	Not compliant (2 violations: HBD > 5; HBA > 10)	PubChem, Rule of Five
